# Isolated Splenic Hydatid Disease

**DOI:** 10.1155/2011/763895

**Published:** 2011-01-18

**Authors:** Alper Dilli, Idil Güneş Tatar, Umit Yasar Ayaz, Baki Hekimoglu

**Affiliations:** ^1^Department of Radiology, Dışkapı Yıldırım Beyazıt Training and Research Hospital, Ministry of Health, Irfan Bastug Caddesi, Diskapi, Altındağ, O6110 Ankara, Turkey; ^2^Department of Radiology, Mersin Women's and Children's Hospital, Ministry of Health, Halkkent, 33240 Mersin, Turkey

## Abstract

Hydatid disease (HD) continues to be a significant health problem in areas where animal husbandry is common but no proper veterinary control exists. The involvement of the spleen in HD is rare, and isolated splenic involvement is even less common. In this case report, we present isolated splenic HD in a 26-year-old female with complaint of abdominal pain, and we discuss some of the clinical aspects of HD. Evaluation of the patient with ultrasonography, computed tomography, and magnetic resonance imaging revealed the presence of an isolated splenic HD as a multivesicular cystic mass located near splenic hilus, measuring 12 × 11 cm. No other organ or system involvement could be demonstrated.

## 1. Introduction

The involvement of the spleen in hydatid disease (HD) is rare, and isolated splenic involvement is even less common. We present isolated splenic HD in a 26-year-old female with complaint of abdominal pain, and we discuss some of the clinical aspects of HD.

## 2. Case Report

A 26-year-old female was referred to our radiology clinic with abdominal pain. The physical examination of the patient revealed splenomegaly. Serology of the patient was consistent with hydatid disease, which helped in the interpretation of the imaging findings. Other laboratory results showed no significant abnormality. We performed abdominal ultrasonography (US), contrast-enhanced abdominal computed tomography (CT), and noncontrast, contrast-enhanced abdominal magnetic resonance imaging (MRI). Chest X-rays and cranial MRI were also obtained to rule out pulmonary and intracranial involvement.

**Figure 1:**
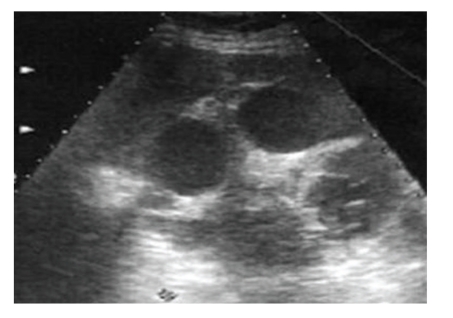
Ultrasonography revealed a multivesicular cystic mass of 12 × 11 cm, in the location of splenic hilus.

In US, we observed a well-defined, multivesicular cystic mass in the location of splenic hilus, measuring 12 × 11 cm. The left kidney was compressed and displaced to the inferolateral position (Figure 1). 

**Figure 2:**
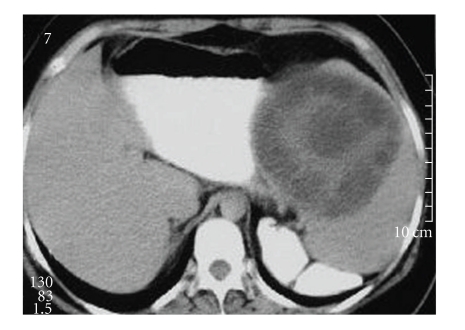
Axial CT with oral and IV contrast shows an unenhanced hypodense mass with well-defined borders in the anterior part of the spleen. Multiple round daughter cysts are seen peripherally within the lesion.

On contrast enhanced CT scans, we detected an unenhancing, hypoattenuating mass located in the anterior portion of the spleen. It had well-defined borders and contained multiple, round, daughter cysts in the periphery of the lesion (Figure 2).

**Figure 3:**
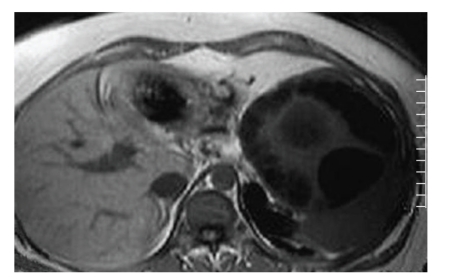
Axial noncontrast T1-weighted MRI shows multivesicular splenic cyst with low-signal-intensity.

**Figure 4:**
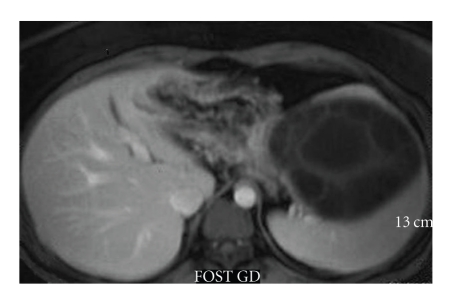
Axial contrast-enhanced T1-weighted MRI shows hypointense multivesicular splenic cyst showing no significant contrast enhancement, except some vague signal increase in the septal parts.

**Figure 5:**
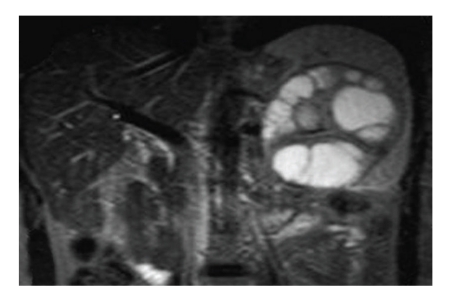
Coronal T2-weighted MRI shows multivesicular cystic lesion located in the middle and lower pole of the spleen, containing hypointense rim and septae.

T1-and T2-weighted MRI revealed a multiseptated cystic mass in the spleen which had a well-defined capsule, the mass showing no significant contrast enhancement, except some vague signal increase in the septal parts (Figures 3, 4, and 5). The hypointense rim sign, characteristic of hydatic cyst, was best seen in the T2-weighted sequence (Figure 5). Pulmonary, hepatic, intracranial, and other tissue involvement could not be demonstrated at the time of diagnosis.

With these imaging findings, final diagnosis was made as hydatid disease of the spleen, and the patient underwent total splenectomy. The lesion was proved to be hydatic cyst pathologically. Besides surgery, the patient was also on medical treatment with albendazole. The patient was in good health in control examinations during the two years followup period.

## 3. Discussion

HD continues to be a significant health problem in areas where animal husbandry is common but no proper veterinary control service is given. It is a unique parasitic disease which can occur in almost any part of the body and demonstrates a spectrum of imaging features that vary according to growth stage, associated complications, and affected tissue [1, 2]. The involvement of the spleen in HD is rare, and isolated splenic involvement is even less common. In our case, only splenic disease was demonstrated without pulmonary, hepatic, intracranial, and any other tissue involvement.

There are two types of *Echinococcus* infections. *E. granulosis* is the more common type, whereas *E. multilocularis* is less common but more invasive, mimicking a malignancy. Radiologic findings range from purely cystic lesions to a completely solid appearance. Hydatid cysts (HCs) are classified on the basis of appearance. Simple cyst with no internal architecture, cyst with daughter cysts and matrix, calcified cyst, or complicated cyst can be observed [3]. 

HCs can be solitary or multiple. US most clearly demonstrates the hydatid sands in purely cystic lesions, as well as floating membranes, daughter cysts, and vesicles. CT is the best modality to detect calcification and internal cystic structure behind calcification. MR imaging is especially helpful in detecting HCs of the central nervous system. 

Liver is the most common site of involvement. Lungs are the second most common sites of hematogenous spread in adults and probably are the most common sites in children (15%–25% of cases). The involvement of the spleen is rare. The reported prevalence of splenic involvement varies from 0.9% to 8% [4]. Splenic HC generally develops via systemic dissemination or intraperitoneal spread from a ruptured liver cyst. Isolated splenic involvement is very uncommon [5]. Splenic HCs are usually solitary, and their imaging characteristics are similar to those of hepatic HCs. Any type of HC can be seen in the spleen [2]. 

In conclusion, being familiar with imaging findings, especially in patients living in countries where this disease is endemic (e.g., parts of South America, the Mediterranean region, the Middle East, Africa, and Australia) and performing serological tests can help establish the diagnosis of HD, as it was in our case.
